# Quality Assessment of Child Care Services in Primary Health Care Settings of Central Karnataka (Davangere District)

**DOI:** 10.4103/0970-0218.62549

**Published:** 2010-01

**Authors:** Rashmi, B Vijaykumar

**Affiliations:** Department of Community Medicine, K. S. Hegde Medical Academy, Nithyanandanagar, Manglore, India; 1 J.J.M. Medical College, Davangere, India

**Keywords:** ARI care, child care, client satisfaction, diarrheal disease care, immunization

## Abstract

**Background:**

Infectious disease and malnutrition are common in children. Primary health care came into being to decrease the morbidity. Quality assessment is neither clinical research nor technology assessment. It is primarily an administrative device used to monitor performance to determine whether it continues to remain within acceptable bounds.

**Aims and Objectives:**

To assess the quality of service in the delivery of child health care in a primary health care setting. To evaluate client satisfaction. To assess utilization of facilities by the community.

**Materials and Methods:**

Study Type: Cross-sectional community-based study. Quality assessment was done by taking 30-50%, of the service provider. Client satisfaction was determined with 1 Immunization and child examination-90 clients each. Utilization of services was assessed among 478 households.

**Statistical Analysis:**

Proportions, Likert's scale to grade the services and Chi-square.

**Results:**

Immunization service: Identification of needed vaccine, preparation and care was average. Vaccination technique, documentation, EPI education, maintenance of cold chain and supplies were excellent. Client satisfaction was good. Growth monitoring: It was excellent except for mother's education andoutreach educational session. Acute respiratory tract infection care: History, physical examination, ARI education were poor. Classification, treatment and referral were excellent. Client satisfaction was good. Diarrheal disease care: History taking was excellent. But examination, classification, treatment, ORT education were poor.

**Conclusion:**

Mothers education was not stressed by service providers. Service providers’ knowledge do not go with the quality of service rendered. Physical examination of the child was not good. Except for immunization other services were average.

## Introduction

All over the world, people seek to improve quality of their life. But most people in developing countries live in overcrowded houses with inadequate sanitation and unsafe water supply. Infectious disease and malnutrition are common in children. Death rate is high and life expectancy is low. To decrease it, primary health care came into being.(1)

Quality assessment depends upon performance of practitioners and contributions of patients and of health care system.(2) Quality assessment is neither clinical research nor technology assessment. It is primarily an administrative device used to monitor performance to determine whether it continues to remain within acceptable bounds.(3) Quality assessment is very much essential to reach the 10 targets of HFA-2020.

Quality medical care, improve in quality of life and eradication of certain diseases are of prime importance. Decentralization of health system is the concept now in the brains of health care professionals. Are we in the path to achieve HFA by 2020? Can we reach the goal? These are the questions that are yet to be answered.

Reproductive and child health service coverage has large differences in various population groups. Special interventions should be undertaken on a priority basis to bridge the gaps so as to achieve millennium development goals in all population groups.(4) The policy implications are that health services and outreach are needed in rural areas in order to increase the level of awareness.(5)

The study is taken to assess extensively the quality of child care in a primary health care set up of Davangere district.

1. To assess the quality of service in the delivery of child health care in a primary health care setting.
2. To evaluate client satisfaction with the government approach.
3. To assess utilization of facilities by the community.

## Materials and Methods

A cross-sectional community-based study conducted in Davangere district with multistage sampling in which the first stage-was stratified sample and second stage was systematic sample within the stratified sample. The district was stratified into taluks. One primary health center and one primary health unit or one community health center (where there is a CHC) from each taluk was selected using random numbers. As district had six taluks, six PHC and five PHU and one CHC were selected. Totally 12 health centers were selected. The sample constituted 12.25% of total health centers of Davangere.(6)

Client satisfaction was determined by systematic random sampling of clients attending the basic health services in randomly selected health centers.(6)

Systematic random sampling of the households of the selected health center area were taken to consider the service approach by asking utilization of services.(6)

Most managers are satisfied with rough estimates. A “***RULE OF THUMB***” is used for rough estimate.

### Quality assessment

Quality assessment was done according to above rule. As in each health centre the population of service providers was <50, so 30-50% of the sample among service provider was taken. So one doctor in each center wherever doctor is present and 20-30% of junior health worker (female) {JHW (F)} and Anganawadi worker in each center were selected.(7)

- There were nine centers with full time doctors. Three centers had no permanent doctors. So the doctor's sample was nine.
- We had 17 JHW (F) in 12 centers instead of 18 JHW(F) because one primary health unit did not have JHW(F).

### Client satisfaction

Client satisfaction was determined for (a) immunization with 90 clients, (b) child examination with 90 clients, so on total we had 180 clients.

### Utilization of services

Utilization of services was assessed using households as the population. As per thumb rule,(7) total house holds sampled were 478 in the district.

Module 6 of Agha Khan Foundation was used for quality assessment of services. The questionnaire was modified according to local needs and was pretested before data were collected.(7) Single observer (first author) did the whole exercise to avoid bias.

Patients who had come to OPD were selected by systematic random sampling and client satisfaction was assessed. Not a single client was interviewed in front of any service provider so that strict confidentiality was maintained.

The assessment of utilization of services was done on the basis of education and socio-economic status rated by B.G. Prasad Classification of 2005 CPI.

### Statistical analysis

1. Proportions of quality under different heading.
2. Chi-square to see association of utilization with socio-economic status and education of the people utilizing hospitals.

Likert's scale was used to grade the quality of service and client's satisfaction:

- <50% → Below average
- 51-65% → Average
- 66-80% → Good
- 81%> → Very good/excellent

## Results

Always immunization was done by JHW (F). An area, where JHW (F) was not present, regular immunization session was not conducted. Identification of needed vaccine, preparation, and care of vaccine was good (66.67%) but 41% of JHF loaded syringe with contamination, 65% kept up cold chain during the session, and 77% of workers checked for expiry. The vaccination technique was excellent (81.86%), but only 5.9% of JHF prepared the area for injection. Sterile needle was used by 88.24% of workers but only 41.2% of them used sterile syringe. EPI education was excellent (83.1%). Maintenance of cold chain and supplies was good (76.47%). Both mothers and service provider's knowledge was excellent. Client satisfaction in immunization is that 100% of clients were satisfied with availability of services, interpersonal quality, professional competence, and skill. 8% of clients were unhappy with duration to wait and fulfillment of health care facility. 30% were unhappy with facilities and equipments, and 20% were unhappy about efficiency to treat.

Growth monitoring and nutrition education were done only by Anganawadi workers. Non-ICDS areas’ growth monitoring was not done. Age calculation and record maintenance were very good (88.89%). The weighing method was also good (68.25%), but children were weighed with the clothes by 78.78% of Anganawadi workers. 89% of workers plotted the growth chart properly. But the malnourished child was never referred to nutritional rehabilitation center. Education to mother on growth monitoring was very poor (12.5%). None were told about the growth card; only 33% of workers told mothers whether child had gained or lost weight compared to earlier session. No JHW(F) told about balanced diet, locally available food, and when the mother have to see her child's weight. 44% of them made recommendation regarding child's feeding and care. Outreach educational sessions were very rarely conducted and importance was not given to these sessions. Supplies were adequate (88.88%). 23% of Anganawadi workers did not have growth charts but all had working scale. Mother's knowledge about growth of her child was poor. Only 41% ad some knowledge; none knew about next weighing session, 31% of mothers knew about gain or lost weight compared to earlier session. Service provider (Anganawadi worker) had very good knowledge (100%).

Both doctors and JHW (F) carry out the service for acute respiratory infection in majority of PHCs. 30% of JHWs (F) were not carrying out this service. History taking was poor (36.3%) as the activity level, ability to drink was not asked by 66.66% of JHW (F), only 5% of health workers asked for ear ache, and 9.5% asked for previous respiratory illness. None asked for treatment administered. Physical examination was also poor (20.41%), temperature was not taken by anyone, 14.3% of workers examined throat and pharynx, and only 29% of health workers counted the respiratory rate. 33.33% of them ausculted the child, but none examined ears nor took temperature. Treatment aspect was good(79.4%) but only 42% of health workers refrained from unnecessary antibiotics. 81% of them administered treatment for fever. ARI education to mothers was again poor (25.2%), none were told about maintaining temperature or feeding habits, only 5% of health workers told about signs of ARI and 38% of them told about completing entire treatment. 52.4% of health workers stressed on next visit within 3 days. Service providers’ knowledge was very good except 25% who did not know about antibiotics prescription and differentiation between cold and pneumonia. Mother's knowledge was poor (36.9%) as found by exit interview of mothers. Supply of antibiotic and thermometer was poor (33.3%).

Both doctors and JHW (F) carry out the diarrheal services in majority of PHC's. In 40% of the PHC visited, JHW (F) were not treating the patients and directing them to doctors. History taking was excellent(79%) except for questions on home treatment which was poor. Examination of the child was poor (23.2%). None of the health provider weighed nor took temperature; 32% of them looked for skin pinch and nutritional status and 52.6% of workers assessed general status. Treatment aspect falls poor (40%) as antidiarrheal was always given by 90% of health workers. Sufficient ORS were not given and degree of dehydration was not determined by 52.63% of health workersbut 63% refrained from antibiotics. ORT education was also poor (24.8%) as no one said about signs of dehydration or danger signs. Nomothers were allowed to ask questions. 58% of health workers told about extra fluid intake and 53% of them explained about ORS preparation whereas 26%of health workers taught about ORS administration and other feeding habits. Supplies were quite good (71.1%). Knowledge of child's mother was very poor (29%) about ORT which was analyzed by the exit interview. Knowledge of service provider was very good (76.3%) But 47.64% of them never knew about child's dehydration grading.

The client satisfaction in child examination was low in interpersonal quality (77.78%) and efficiency to treat (83.33%). 13% of clients had problem with professional competence and skill, 9% had problem with duration to wait and fulfillment of health care facility, and 100% of clients were satisfied with availability of services, facilities, and equipments.

### Utilization of services in child care

Education of the clients by itself has a significant (*P* < 0.05) association for the people's preference to private health sector for child care which is very high among SSLC + people (47.75%). But interestingly graduates have less preference (36.21%) than SSLC + and they prefer first public and then private (64%). But considering people with nil education and some education (31%), graduates have higher preference to private regarding child care. High socio-economic status is significantly associated (*P* < 0.05) with peoples preference to private heath sector (43.78%) and it goes on decreasing among people with low socio-economic status (26.61%).

## Discussion

Identification of vaccine and preparation of vaccine was good were it was graded as excellent in the Sunderlal *et al*.'s(8) study. Syringes were loaded with contamination (41%) much higher than stated by the Sunderlal *et al.* 's(8) study. Very few (41.2%) used disposable syringe and needle, which is similar to that of earlier studies.(8,9) The problem was in preparing the site of injection as only 5.9% did it. Documentation and EPI education both were excellent similar to the Sunderlal *et al*.(8) study. Questions were not allowed by service provider which led to non-interaction between provider and mother similar to earlier(10) study. Waiting duration was long in immunization for few mothers, on an average 30;min was acceptable but, few mothers had to wait for 1-2 h. Facilities and equipments were average for immunization because of which people were unhappy especially on the non-availability of disposable needle, syringes, and vaccines like tetanus toxoid and BCG in certain places at certain time., 20% of clients felt many a times their queries were not answered and sometimes Thursday sessions of immunization were missed out.

In our study children's age were measured correctly similar to all earlier(11–13) studies. No children were referred for nutritional counseling and only one-third of mothers were told about weight of the child, which was also similar to earlier studies.(11,14) Outreach educational sessions were very poor as found in an earlier study.(11) As a result, mothers had very poor knowledge of the growth monitoring which definitely hampers the nutritional status of children, unless the caregiver, is oriented toward the growth monitoring, and the available food to improve child's growth, all the exercises done by the health worker is of vain. Though right weighing was carried out proper referral and mother's education needed to be stressed on.

In the history taking of ARI, almost all asked about the presence of fever and cough. Treatment history and past history were never or poorly asked, and 63% clients were asked to refrain from antibiotics similar to earlier studies.;(12) NFHS-2 says most often clients are treated with antibiotics and cough syrup, so according to our study the unnecessary use of antibiotics had been cut down. Physical examination was also found to be poor in our study but better than the Agarwal *et al*. study.(11) The study says none had told about extra fluids, only 4.16% told about severe ARI and none were asked for queries similar to earlier studies.(11,14) Mothers knowledge about ARI was poor and only 9.52% of mothers knew about danger signs, whereas in Bojalil *et al*.(14) study 80% knew about the danger signs. The performances of the poor quality of service do not go with knowledge of service provider which was good. It looks like it a just careless feeling toward the patient or inability to transfer the knowledge to patient. But supplies were average (which might have also affected the quality of care.

History taking in diarrheal disease was good unlike an earlier study.(11) Examination of child was poor as the earlier study.(11) Assessment of general status was done by 30.8% in the Agarwal *et al*.(11) study, whereas in our study 52.64% of them did it. CDD(15) surveys also have shown concern about low quality treatment practices and reported inadequate assessment of status of dehydration, observed error in the performance of health providers while determining severity of dehydration in children. ORT education was poor similar with Agarwal *et al*. study.(11) Preparation of ORS was demonstrated by 52.6% health workers, and 26.32% of them told how often to feed which is less as compared to the earlier study.(11) Same as the earlier study, none were told about danger signs. Mothers knew very less about ORS (29.95%), even the service provider was not competent (76.32%) and even CDD(15) surveys suggested that health workers were lacking in technique of classifying the illness and health education and so missed the opportunity to educate mothers on various diarrhea case management issue which in long run can change the community practices and can save the life of many children in future.

9% of clients were unhappy about waiting time, as there was no importance for sick child. 22.22% were unhappy with interpersonal quality, i.e. no proper answer to queries and not allowed to ask questions. 13.33% were not satisfied with professional competence, as they felt children need specialist care and service provider was not competent to cure the disease.

Utilization of services is almost equal among private and public unlike Nougtara *et al*.(16) where utilization was 13% in the public health sector. In earlier studies(17,18) clients rated that the private sector was better than the public service. But in our study almost equal distribution was seen in both the sectors. When the utilization was compared with the education of the people and socio-economic status, it was a very obvious finding that socio-economic status of classes I and II had more inclination toward the private sector and educated people had less inclination toward the public sector.

### Limitations

This quality assessment, client satisfaction, and utilization of services everything is done by thumb rule as sample size, so this gives rough estimate. Sample size should be increased to get an accurate estimate. As this is a primary study, rough estimate was considered.

## Conclusion

The immunization process was excellent except for few drawbacks and interaction with mothers. Client satisfaction in regard to immunization was excellent in all aspects except satisfaction for efficiency to treat which was average due to unanswered queries and missed sessions. Utilization of services was 100% without any effect of SES or education. Growth monitoring was excellent except for mother's education. Service provider knowledge was very good, but mother's knowledge was poor.

History, physical examination, and ARI education were poor. Classification, treatment, and referral were excellent but supplies were average. Knowledge of service providers was very good but still quality was average. History taking in diarrheal diseases was excellent. But examination, classification, treatment, and ORT education were poor. Supplies and service provider knowledge was good. Child's mother had a poor knowledge. Client satisfaction was average with interpersonal quality rated to be poor. Utilization of service was 64.50% dependent on socio-economic status and education.
